# Expression Profile Analysis of Long Non-coding RNA in OVX Models-Derived BMSCs for Postmenopausal Osteoporosis by RNA Sequencing and Bioinformatics

**DOI:** 10.3389/fcell.2021.719851

**Published:** 2021-09-30

**Authors:** Huijie Gu, Zhongyue Huang, Kaifeng Zhou, Guangnan Chen, Chong Bian, Jun Xu, Xiaofan Yin

**Affiliations:** Department of Orthopedics, Minhang Hospital, Fudan University, Shanghai, China

**Keywords:** osteoporosis, lncRNA, BMSCs, OVX model, differentially expressed, NONMMUT0961501

## Abstract

Osteoporosis (OP) has the characteristics of a systematically impaired bone mass, strength, and microstructure. Long non-coding RNAs (lncRNAs) are longer than 200 nt, and their functions in osteoporosis is yet not completely understood. We first harvested the bone marrow mesenchymal stem cells (BMSCs) from ovariectomy (OVX) and sham mice. Then, we systematically analyzed the differential expressions of lncRNAs and messenger RNAs (mRNAs) and constructed lncRNA–mRNA coexpression network in order to identify the function of lncRNA in osteoporosis. Totally, we screened 743 lncRNAs (461 upregulated lncRNAs and 282 downregulated lncRNAs) and 240 mRNAs (128 upregulated and 112 downregulated) with significantly differential expressions in OP compared to normal. We conducted Gene Ontology (GO) and Kyoto Encyclopedia of Genes and Genomes (KEGG) functional analyses to investigate the functions and pathways of the differential expression of messenger RNAs (mRNAs), a coexpressed network of lncRNA/mRNA. Quantitative PCR (qPCR) validated that the expressions of NONMMUT096150.1, NONMMUT083450.1, and NONMMUT029743.2 were all downregulated, whereas NONMMUT026970.2, NONMMUT051734.2, NONMMUT003617.2, and NONMMUT034049.2 were all upregulated in the OVX group. NONMMUT096150.1, as a key lncRNA in OP, was identified to modulate the adipogenesis of BMSCs. Further analysis suggested that NONMMUT096150.1 might modulate the adipogenesis of BMSCs via the peroxisome proliferator-activated receptor (PPAR) signaling pathway, AMPK signaling pathway, and the lipolysis regulation in adipocyte and adipocytokine signaling pathway. Our study expands the understanding of lncRNA in the pathogenesis of OP.

## Introduction

Osteoporosis (OP) has the characteristics of a systematically impaired bone mass, strength and microstructure, which generates fracture risk and exhibits an association with substantial pain, disability, and even death (Kanis et al., 2019). Postmenopausal osteoporosis (PMOP) is the most common primary osteoporosis (POP) and results from estrogen deficiency (Rosen, 2000). Several studies indicate that the imbalance differentiation of BMSCs plays a basic role in PMOP. Nonetheless, it is yet elusive toward the underlining mechanisms toward PMOP (Noh et al., 2020).

lncRNAs, a sort of non-coding RNAs with > 200 nt, are largely reported to have an association with various diseases, including osteoporosis (He et al., 2020; He and Chen, 2021). lncRNA itself does not encode protein and regulates gene expression at epigenetic, transcription, and posttranscriptional levels (Dykes and Emanueli, 2017; Wang et al., 2019). lncRNA regulates the expression of mRNA encoding protein by binding to microRNAs (miRNAs) of specific sequences, so lncRNA is called miRNA sponge (Yoon et al., 2014). Li et al. (2018) identified that lncRNA Bmncr mediates age-related bone loss via reversing the age-related alteration between osteogenesis and adipogenic differentiation of BMSCs. Yin et al. (2021) reported that lncRNAs (AK039312 and AK079370) exerted an inhibition effect on the differentiation of osteoblast and the formation of bone by suppressing osteogenic transcription factors via targeting miR-199b-5p, then activating GSK-3β and in-depth inhibiting the Wnt/β-catenin pathway. Knockdown of LNC_000052 could promote osteogenesis and inhibit apoptosis of BMSCs via the PI3K/Akt signaling pathway (Li et al., 2020). Increasing study indicates that lncRNAs exhibit importantly in BMSCs differentiation and osteoporosis. Nevertheless, it is not clear on the roles of most lncRNAs in osteoporosis.

Here, we utilized RNA-sequencing to identify the differentially expressed lncRNAs (DElncRNA) and mRNAs (DEmRNAs) of BMSCs from sham and OVX mice. Subsequently, we constructed a protein–protein interaction (PPI) network of DEmRNAs and coexpressed networks of DElncRNA/DEmRNA and analyzed the function of networks by GO and KEGG. We determined sectional DElncRNAs expression by quantitative real-time PCR (qRT-PCR). Our findings gave a new insight into clarifying lncRNAs’ regulatory role in PMOP.

## Materials and Methods

### Ovariectomy Mouse Model

The animal experimental protocols got the approval of the Animal Care and Experiment Committee of Fudan University. Six-week-old C57BL female mice (about 20–24 g of weight) were obtained from the Shanghai Model Organisms Center (Shanghai, China). No obvious difference existed in mice’s weight at the start. Twelve mice received an ovariectomy operation (OVX), while another 12 mice received sham operation, after anesthesia with intraperitoneal injection of pentobarbital sodium (50 mg/kg body weight). We executed ovariectomy as previously described (Gu et al., 2020).

### Bone Mineral Density Measurement and Micro-CT Analysis

At 6 weeks postoperation, we harvested the left femurs to determine the bone mineral density (BMD) via dual-energy X-ray absorptiometry (DXA; GE Healthcare, Piscataway, NJ, United States). Then, we fixed the left femur with 4% paraformaldehyde for 24 h and analyzed it by SkyScan-1176 microcomputed tomography (μCT) (Bruker microCT, Kontich, Germany). The trabecular bone volume/tissue volume ratio (BV/TV) of the distal femur was measured.

### mBMSCs Culture

According to previous reports, bone marrow was flushed from the femur and tibia and then isolated mBMSCs (Gu et al., 2020). Taken briefly, at 6-week postoperation, the mice were euthanized. We harvested the femur and tibia and removed the epiphyses of individual bones. Bone marrow was washed from the diaphysis by medium containing low-glucose Dulbecco’s modified Eagle’s medium (DMEM) (LG-DMEM; Gibco BRL, United States), 10% fetal bovine serum (FBS) (Gibco BRL, United States), 2 mM glutamine, 1% streptomycin/penicillin. We obtained cell suspension by repeatedly aspirating cells with the use of 20-gauge needle. Cells (1 × 10^6^) with indicated culture medium were inoculated in 60 mm culture dish under 37°C incubator containing 5% CO_2_. We changed the medium twice 1 week and utilized the cells with well status after three or five passages.

### RNA Extraction, RNA-Sequencing, and Analysis of Differentially Expressed Genes

MiRNA isolation kit (Ambion, Austin, TX, United States) was applied to extract the whole RNA from BMSCs of three mice in an individual group. TruSeq stranded total RNA with Ribo-Zero Gold (Illumina, San Diego, CA, United States) was employed to generate the libraries as the manual described. RNA was sequenced on the Illumina sequencing platform (HiSeq^TM^2500; Shanghai Oebiotech Co., Ltd., Shanghai, China). Paired-end reads (125 bp/150 bp) were generated. Then, lncRNAs and mRNAs with the differential expression between the sham and OVX mice were identified by DESeq software packages.^1^ The differentially expressed genes were identified with the criteria of fold change > 2; *p* < 0.05. TBtools was used to analyze the distribution of DElncRNAs and DEmRNAs on chromosomes (Chen et al., 2020).

### Functional Annotation of DEmRNA and DElncRNA-Correlated mRNAs

The Gene Ontology (GO) was conducted to explore DEmRNAs and lncRNA coexpressed mRNAs’ biological functions. Kyoto Encyclopedia of Genes and Genomes (KEGG) was employed to uncover the pathways of DEmRNAs and lncRNA coexpressed mRNAs. *p* < 0.05 means there is a statistically significant difference regarding the GO terms and pathways.

### DElncRNA–DEmRNA Coexpression Analysis

DElncRNA–DEmRNA coexpression analysis was taken to investigate DElncRNAs’ roles in osteoporosis. We analyzed DElncRNAs and DEmRNAs referring to the criteria of fold change > 2, *p* < 0.05. DElncRNA–DEmRNA pairs with correlation coefficient ≥ 0.98 and *p* < 0.05 were selected in the analysis. We visualized the coexpression network utilizing the software Cytoscape 3.7.0, and we measured the nodes’ centrality in the coexpression network by K-core analysis.

### DElncRNAs Validation

Seven DElncRNAs were validated by qPCR. The cDNAs were reversed transcribed from RNA utilizing the NCode^TM^ EXPRESS SYBR^®^ GreenER^TM^ miRNA qPCR kit (Invitrogen, Carlsbad, CA, United States). We conducted qPCR reaction on the GeneAmp PCR system 9600 (Perkin Elmer, United States). All primers were obtained from Sangon Biotech (Shanghai, China), and their sequences are shown in Table 1. The relative lncRNAs expression was obtained after normalization to β-actin. 2^–Δ^ ^Δ^ ^*Ct*^ method was applied to calculate the expression level of each lncRNA.

**TABLE 1:** Primers for qRT-PCR in the experiment.

Gene symbol	Forward primer	Reverse primer
NONMMUT096150.1	GCCGTCCTTACAGGAGTGAA	GGTTTCCGCACGGGATGTA
NONMMUT083450.1	TTTGAGGGCCTCTCTAGCCT	CAGACCTAGCAGATGGAGCG
NONMMUT029743.2	GAGCAAGTTGTGTGTGCCAG	CAAAACCCTGACCTTGCAGC
NONMMUT026970.2	TTCTCCCTCGTTACCCGACT	CCTCAACGGAGACACACTCC
NONMMUT051734.2	ACTGCCTTTCCTTGTCCCTG	TGTGCTGTGAACCAAGCTGA
NONMMUT003617.2	TTAGAAGCATCCCGTGGTCC	TTCTCAGACTGTCCTCGGGT
NONMMUT034049.2	CGCACCATTGCACTTGTTGA	AACAAAGCCTGCCTCTCTCC
β-actin	ATCATGTTTGAGACCTTCAA	CATCTCTTGCTCGAAGTCCA

### Statistical Analysis

All representative data was analyzed by SPSS 22.0 (IBM Corp., Armonk, NY, United States). The data were shown as the mean ± standard deviation (SD). The difference existing in two and more groups were determined by Student’s *t*-test and one-way analysis of variance (ANOVA), respectively. The fold change of each lncRNA expression was shown after calculation by the 2^–Δ^ ^Δ^ ^*Ct*^ method. *p* < 0.05 means that there is a significant difference in compared groups.

## Results

### Ovariectomy Mice Model Evaluation

**FIGURE 1:**
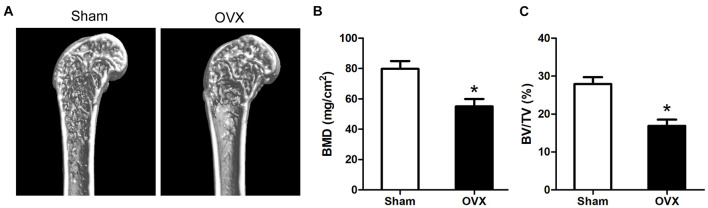
Evaluation of the OVX mouse model. **(A)** The 3D micro-CT images of the distal and middle femurs. **(B)** OVX mice had lower BMD as compared to the controls. **(C)** BV/TV of femurs were measured by micro-CT. *p < 0.05 vs Sham group.

We evaluated the OVX mice model as the methods described before. We harvested left femurs after 6 weeks of operation and tested them. A reduced bone formation was indicated by micro-CT in the OVX group (Figure 1A). The BMD and bone volume (BV)/total volume (TV) of OVX mice were shown to reduce in comparison with the sham group (Figures 1B,C).

### DElncRNAs and DEmRNAs in Bone Marrow Mesenchymal Stem Cells of Ovariectomy Mice

**FIGURE 2:**
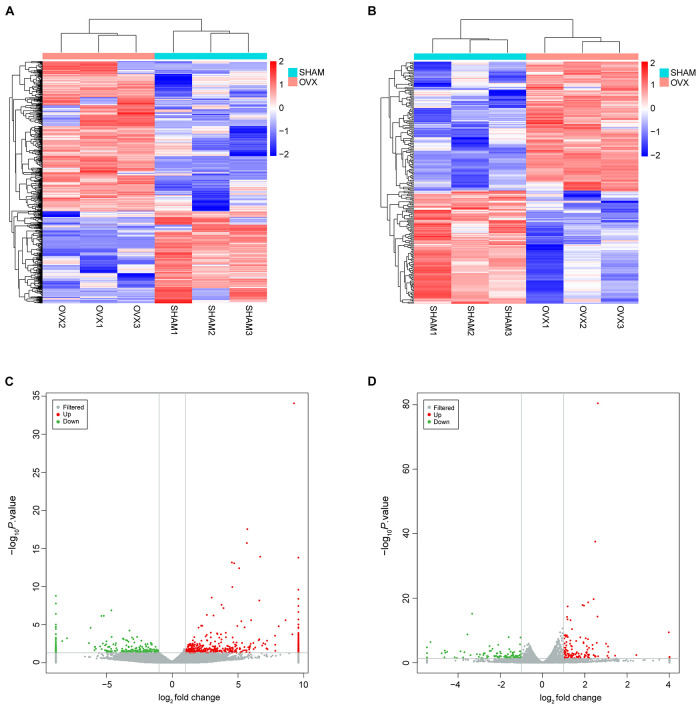
Expression profiles of lncRNAs and mRNAs. **(A)** Cluster analysis of differentially expressed lncRNAs. **(B)** Cluster analysis of differentially expressed mRNAs. Red indicates increased expression, and blue denotes decreased expression. **(C)** The volcano plot of differentially expressed lncRNAs. **(D)** The volcano plot of differentially expressed mRNAs.

The gene expression variation between OVX and sham groups was assessed by heat map analysis and volcano plots (Figure 2). In this study, the DElncRNAs and DEmRNAs were identified. In total, 743 DElncRNAs were identified in OVX mice, including 461 upregulated lncRNAs, and 282 downregulated lncRNAs; the top 10 DElncRNAs were NONMMUT026970.2, NONMMUT010789.2, NONMMUT016319.2, NONMMUT0 51734.2, NONMMUT036562.2, NONMMUT015053.2, NON MMUT069358.2, NONMMUT034071.2, NONMMUT038202.2, and NONMMUT026990.2 (Supplementary Table 1). Two hundred forty DEmRNAs were identified in OVX mice, including 128 upregulated and 112 downregulated; the top 10 DEmRNAs were Scd1, Plin1, Cyp2f2, Cfd, Cidec, Thrsp, Fabp4, A2m, Glb1l2, and Cxcl14 (Supplementary Table 2).

**FIGURE 3:**
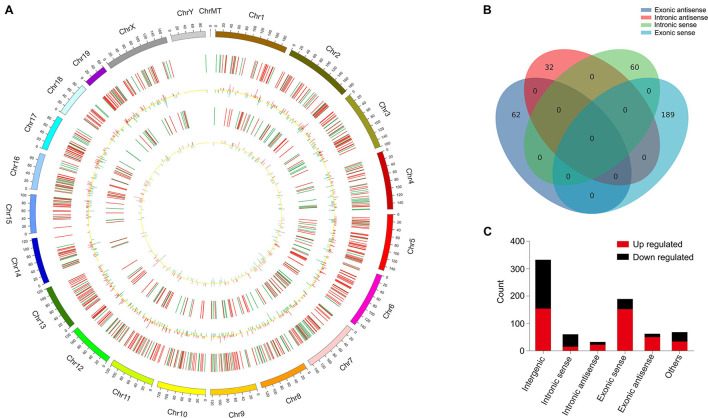
Identification of differentially expressed long non-coding RNAs (lncRNAs) in OVX. **(A)** Circos plot showing lncRNAs and mRNAs on mouse chromosomes. From the outside in, the first layer of the Circos plot is a chromosome map of the mouse genome. The second layer represent the distribution of DElncRNA in mouse chromosomes. Red indicates increased expression, and blue denotes decreased expression. The third layer represent the mean expression values of significantly differentially changed lncRNAs in OVX. The fourth circle represent the distribution of DEmRNA in mouse chromosomes. The fifth layer represent the mean expression values of significantly differentially changed mRNAs in OVX. Red indicates increased expression, and blue denotes decreased expression. **(B)** Venn diagram presents overlapping relationships, and the numbers indicate lncRNA counts. **(C)** Types and counts of differently regulated lncRNAs classified into six categories according to the genomic loci of their neighboring genes.

These DElncRNAs and DEmRNAs were distributed in all chromosomes including chromosomes X and Y (Figure 3A). The DElncRNAs (totally 743) were classified into six categories: 332 were intergenic, 60 were intronic sense, 32 were intronic antisense, 189 were exonic sense, 62 were exonic antisense, and 68 were others. There were no overlaps between the four categories, including intronic and antisense, and exonic sense and antisense (Figure 3B). Intergenic and exonic sense lncRNAs constituted the largest number in all DElncRNAs and comprised 44.7 and 25.4%, respectively (Figure 3C).

### Functional Analysis of DEmRNAs

**FIGURE 4:**
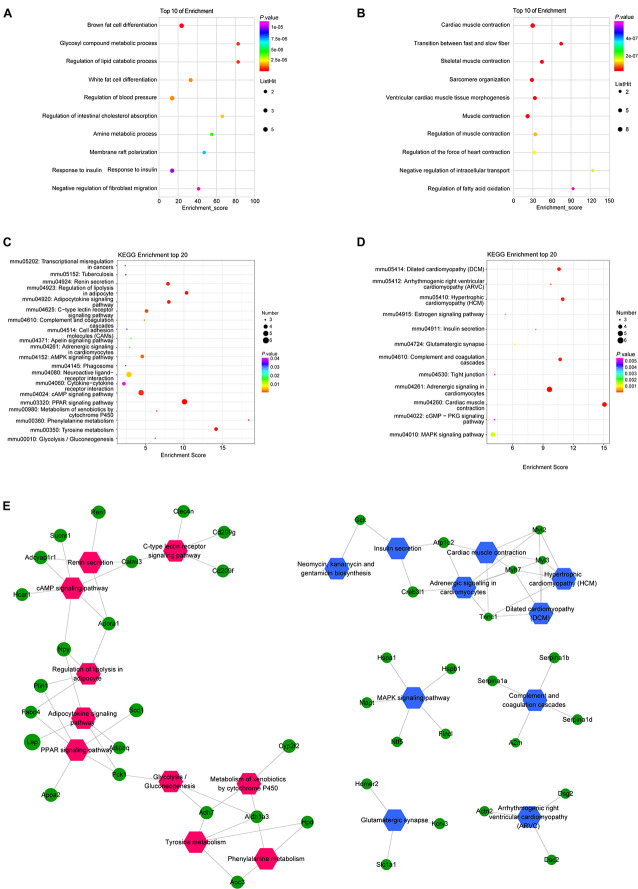
Gene Ontology (GO) and Kyoto Encyclopedia of Genes and Genomes (KEGG) pathway analyses of mRNAs in OVX. GO annotations of up **(A)** and down **(B)** regulated mRNAs with top 10 enrichment scores of biological processes. KEGG pathway enrichment analysis of up **(C)** and down **(D)** regulated mRNAs with top 20 enrichment scores. Interaction and overlapping of associated molecules among significant pathways **(E)**. Hexagons represent the most significantly enriched pathways; ellipses indicate mRNAs that act as the link hinge between the pathways.

**FIGURE 5:**
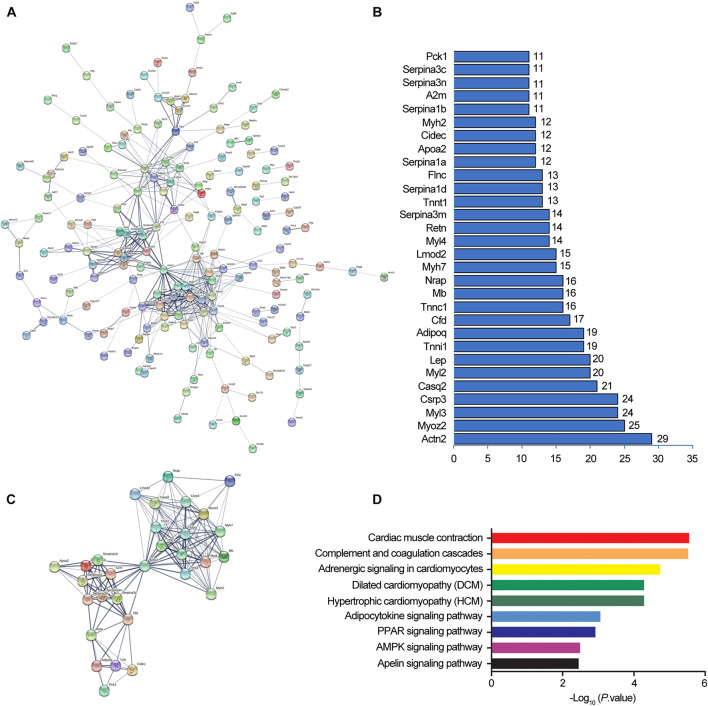
Protein-protein interaction networks by Search Tool for the Retrieval of Interacting Genes/Proteins (STRING). STRING software constructed the differentially expressed mRNAs (fold change ≥ 2, and *P* < 0.05) network based on protein-protein interactions **(A)**. A confidence score that calculated for all protein interactions based on experimentally and computationally interaction was set as the medium (>0.4). Thirty top k-score genes involved in the network **(B)**. Thirty top k-score genes constitute the subnetworks **(C)**. KEGG pathway annotations of the network **(D)**.

In order to forecast the function of DElncRNAs in osteoporosis, we first analyzed the functions of DEmRNAs via GO and KEGG pathway analyses, which provided a clue about osteoporosis. The significantly enriched GO targeted by mRNAs with up- and downregulation primarily participated in cardiac muscle contraction, skeletal muscle contraction, the transition between fast and slow fiber, and brown fat cell differentiation (Figures 4A,B and Supplementary Tables 3, 4). The KEGG pathway analysis revealed that mRNAs with up- and downregulation were mostly enriched in the peroxisome proliferator-activated receptor (PPAR) signaling pathway and cardiac muscle contraction, respectively (Figures 4C,D and Supplementary Tables 5, 6). The top 20 significantly enriched pathways were employed to construct a pathway network to expound the important pathways in the development of osteoporosis. The PPAR signaling pathway, lipolysis regulation in adipocyte, adipocytokine signaling pathway, and mitogen-activated protein kinase (MAPK) signaling pathway, which were involved in osteogenesis and adipogenesis of BMSCs, were included in the network and exchanged with each other (Figure 4E). We constructed DEmRNAs network by Search Tool for the Retrieval of Interacting Genes/Proteins (STRING) database to further unearth genes’ function at the protein level. The interactions among DEmRNAs were evaluated by the medium confidence score (0.4). One hundred sixty-eight nodes and 440 edges were included in the network (Figure 5A). The k-core of nodes was calculated, and the 30 highest k-core DEmRNAs are shown in Figure 5B. The top 10 hub DEmRNAs include Cfd, Adipoq, Tnni1, Lep, Myl2, Casq2, Csrp3, Myl3, Myoz2, and Actn2. These 30 DEmRNAs constituted two important subnetworks, which were enriched in the PPAR signaling pathway, adipocytokine signaling pathway, and AMPK signaling pathway, involving in the differentiation of BMSCs (Figures 5C,D).

### lncRNA–mRNA Coexpression Network in Osteoporosis

**FIGURE 6:**
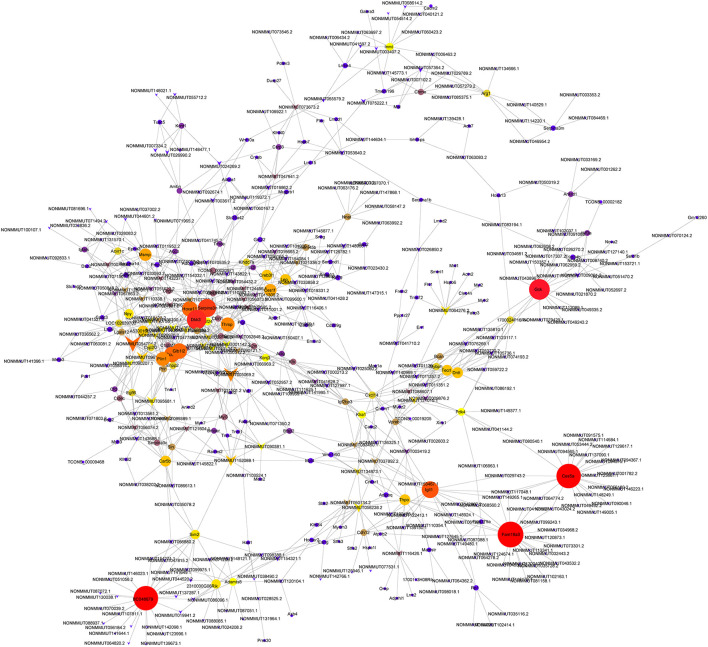
Coexpression networks constructed by weighted correlation network analysis. Circle, mRNA; diamond, long non-coding RNA; line, correlative relationship. Different sizes and colors represent the corresponding k-core scoring.

We performed lncRNA–mRNAs coexpression analysis and constructed the coexpression networks to identify the function of lncRNAs in osteoporosis. The coexpression network of lncRNA–mRNA (correlation coefficient ≥ 0.98) was constructed using Cytoscape3.5.1 (Figure 6) and included 608 network nodes and 1,125 connections. The k-core of network nodes was calculated, and the results (k-core ≥ 5) are shown in Supplementary Table 7. The three highest k-core lncRNAs were NONMMUT054704.2, NONMMUT005069.2, and NONMMUT096150.1.

### Functional Analysis of the Coexpression Network

**FIGURE 7:**
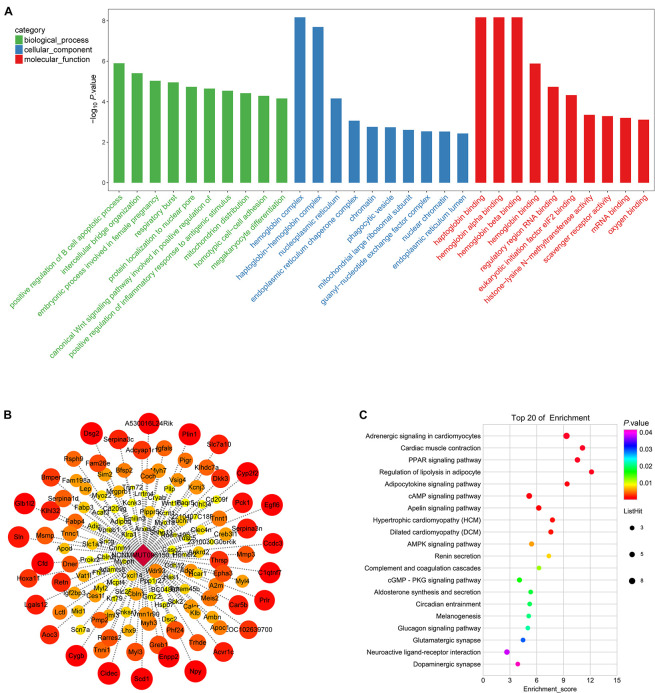
Gene Ontology (GO) annotations for long non-coding RNAs (lncRNAs) in in OVX. **(A)** The figure shows top 10 most enriched GO for DelncRNA in ontologies of biological processes, cellular component, and molecular function. **(B)** mRNAs that coexpressed with lncRNA RP11-222K16.2. Weighted correlation network analysis based on Pearson’s correlation was used to estimate the correlation coefficient between the lncRNA and coding genes, and the soft threshold was set at 0.8. **(C)** KEGG pathway annotations of the lncRNA NONMMUT096150.1.

To elucidate DElncRNA’s biological role, we illustrated mRNAs coexpressed with lncRNA by GO (Supplementary Table 8) and KEGG (Supplementary Table 9). The top 30 GOs are shown in Figure 7A. GO terms, such as positive regulation of inflammatory response to an antigenic stimulus, protein localization to nuclear pore, and megakaryocyte differentiation, were significantly enriched. The enriched signaling pathways (*p* < 0.05) can be a reflector of lncRNAs’ function in osteoporosis. NONMMUT096150.1 was shown as an example by flow chart analysis (Figures 7B,C). mRNAs that coexpressed with NONMMUT096150.1 are shown in Figure 7B. The function of NONMMUT096150.1 was annotated by their connections with mRNAs. The top 20 enriched KEGG pathways were identified in these DEmRNAs coexpressed with NONMMUT096150.1 (*p* < 0.05, Figure 7C), including PPAR signaling pathway, lipolysis regulation in adipocyte, and adipocytokine signaling pathway. The functional annotation indicates that the lncRNA may have multiple functions in osteoporosis.

### Validation of Differentially Expressed lncRNA in Ovariectomy vs. Sham Control

**FIGURE 8:**
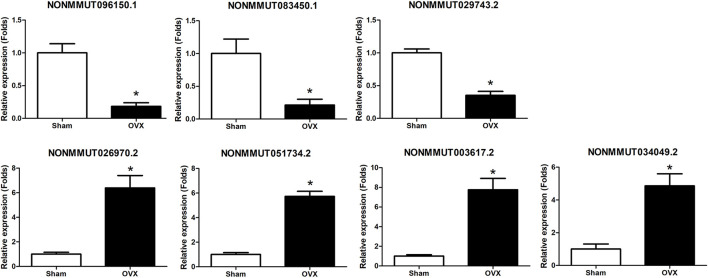
Validation of DElncRNA expression by qRT-PCR between OVX mice and controls. *p < 0.05 vs Sham group.

Seven DElncRNAs were validated by qPCR in this study. We chose three lncRNAs with downregulated expression in the OVX group, namely, NONMMUT096150.1, NONMM UT083450.1, and NONMMUT029743.2, and four lncRNAs with upregulated expression in OVX group, namely, NONMMUT026970.2, NONMMUT051734.2, NONMMUT003 617.2, and NONMMUT034049.2 for qPCR validation. Consistent with the sequencing findings, we verified that NONMMUT096150.1, NONMMUT083450.1, and NONMMUT029743.2 were all downregulated, whereas NONMMUT026970.2, NONMMUT051734.2, NONMMUT003 617.2, and NONMMUT034049.2 were all upregulated in the OVX group, as shown in Figure 8. Thus, the qPCR results fully supported the reliability of lncRNA expression profiles of OVX by sequencing analysis.

### lncRNA NONMMUT096150.1 Involved in the Adipogenesis Through Transcription Factor Association Analysis

**FIGURE 9:**
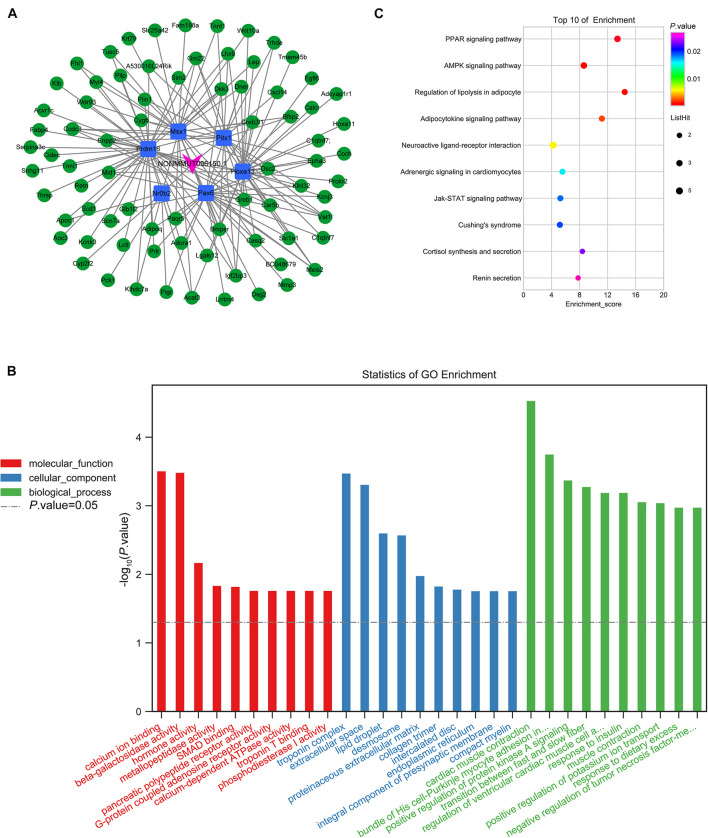
Coexpression network of NONMMUT096150.1-TFs-mRNAs in the OVX mouse model. **(A)** NONMMUT096150.1-TFs-mRNAs networks in OVX mice. **(B)** The top 10 GO enrichment analyses of cellular component, molecular function and biological process. **(C)** KEGG pathway annotations of the network.

The GO and KEGG analysis showed that NONMMUT096150.1 was involved in the adipogenesis of BMSCs. Furthermore, the qPCR results validated that NONMMUT096150.1 was downregulated in BMSCs from OVX mice. In order to understand how lncRNA NONMMUT096150.1 functioned in osteoporosis, we had the deeper analysis of NONMMUT096150.1 through transcription factor association analysis. In this study, we identified five coexpressions between NONMMUT096150.1 and transcription factors (TFs), including Hoxa13, Pax6, Nr0b2, Prdm16, Msx1, and Pitx1, and 156 relations between TFs and mRNAs. Figure 9A shows that Cytoscape 3.5.1 was employed to construct the coexpression network of NONMMUT096150.1-TFs-mRNAs. The mRNAs in this network were annotated by GO and KEGG pathway analysis to investigate in-depth NONMMUT096150.1’s functions in osteoporosis. GO analysis showed that GO terms (calcium ion binding, troponin complex, and cardiac muscle contraction) were greatly enriched. The top 30 terms are shown in Figure 9B. A total of 17 enriched KEGG pathways exhibited great enrichment (*p* < 0.05, Supplementary Table 10). The top 10 pathways are displayed in Figure 9C, including the PPAR signaling pathway, AMPK signaling pathway, and lipolysis regulation in adipocyte and adipocytokine signaling pathway. The functional annotation indicates that the lncRNA NONMMUT096150.1 may function as an adipogenesis regulatory in OP.

## Discussion

The imbalanced bone formation and resorption induced by various factors, such as genetic interaction, epigenetic, and environmental, was the basic pathogenesis of OP (Bristow et al., 2014). Several studies have explored the molecular mechanisms of OP; however, the role of lncRNAs in OP has just begun to be understood, and most of them have not yet been investigated. Here, we identified the DElncRNAs and DEmRNAs between OVX and sham mice using RNA-sequencing. Subsequently, we constructed the coexpression network of lncRNA/mRNA and performed GO and KEGG pathway analyses to uncover lncRNAs’ biological functions in the pathogenesis of OP. We verified the primary lncRNAs expression by qPCR and also explored their function by functional experiments. Our work indicates a coexpression lncRNA/mRNA network involved in the development of OP, indicating that lncRNAs functioned importantly.

In the present study, we identify 240 DEmRNAs in OVX, compared with control mice, via RNA-seq. Some of the DEmRNAs are reported to be involved in OP in previous studies. Adig, as an adipocyte-enriched transmembrane protein, is essential for adipogenesis (Kim et al., 2005). The deficiency of Adig-reduced plasma leptin levels regulated by fat mass and exposed an impairment of leptin secretion from adipose explants (Alvarez-Guaita et al., 2021). Fabp3 is reported to promote adipogenesis and inhibit osteogenesis of BMSCs. Accumulation of Fabp3 leads to the occurrence of OP through impaired osteogenesis of BMSCs, while knockdown of Fabp3 reduces bone loss in aged mice (Liu et al., 2020). DKK3 is reported to negatively regulate the osteogenesis of rat dental follicle cells (Zhang et al., 2017). Ambn is reported to modulate the osteogenic capacity of BMSCs (Stakkestad et al., 2017). Lu et al. (2016) found that Ambn stimulates long bone growth and increases osteogenic gene expression and proliferation of osteoblast via modulating the production of extracellular matrix and the properties of cell adhesion in the long bone growth plate. The GO and KEGG pathway analyses indicated that the DEmRNAs participated in the OP pathogenesis. The upregulated DEmRNAs were involved in adipocytokine signaling pathway, apelin signaling pathway, AMPK signaling pathway, and PPAR signaling pathway, while the downregulated DEmRNAs were involved in the estrogen signaling pathway and MAPK signaling pathway. There was crosstalk between each signaling pathway via several mRNAs. Furthermore, we constructed the PPI network of DEmRNAs via STRING. The top 30 highest k-core nodes were selected and constructed to the subnetwork. The KEGG analyses of this subnetwork revealed that the 30 nodes took part in adipogenesis signaling pathways, including adipocytokine signaling pathway, PPAR signaling pathway, AMPK signaling pathway, and apelin signaling pathway. Previous studies have shown that ginsenoside Rg3 reduces osteoporosis caused by ovariectomy through the AMPK/mTOR signaling pathway (Zhang et al., 2020). Exosomes derived from bone marrow mesenchymal stem cells promote the proliferation of osteoblasts through the MAPK pathway to improve osteoporosis (Zhao et al., 2018). Isopsoralen regulates PPAR-γ/Wnt to inhibit oxidative stress in osteoporosis (Wang et al., 2018). In summary, DEmRNAs may participate in the development of osteoporosis by regulating related signal pathways.

We identified 743 DElncRNAs in OVX mice, with 461 upregulation, and 282 downregulation. The biological functions of lncRNAs are closely related to the mRNAs that they regulate. We constructed the coexpression network of DElncRNAs and DEmRNAs and analyzed the function by bioinformatics. The results indicated that this coexpression network was involved in the differentiation of BMSCs. Four lncRNAs might have been validated in BMSCs of OVX. Among them, NONMMUT096150.1 gained our attention, as its k-core was high in the coexpression network, and its expression is downexpressed in OVX. One hundred thirty-nine mRNAs coexpressed with NONMMUT096150.1; furthermore, KEGG analysis showed that the lncRNA may regulate the adipogenesis of BMSCs. In our previous study, we have indicated that the adipogenesis of BMSCs in OVX mice is upregulated (Gu et al., 2020).

The lncRNAs mediated mRNAs expression regulation by modulating the expression and/or function of TFs (Wang et al., 2021). Therefore, we further define the function of lncRNA NONMMUT096150.1 through transcription factor association analysis. The coexpression network of NONMMUT096150.1-TFs-mRNAs is constructed. There are six TFs, including Hoxa13, Pax6, Nr0b2, Prdm16, Msx1, and Pitx1, in this coexpression network. Overexpression of Hoxa13 stimulates the osteogenic differentiation of MC3T3-E1 via increasing the protein level of β-catenin (Li et al., 2021). Pax6 is reported to be a negative regulator of osteoclastogenesis and attenuates the OC differentiation via activation of Acp5 gene (Kogawa et al., 2013). Another study shows that p38/β-catenin/Pax6 axis could inhibit osteoclastogenesis (Jie et al., 2019). Nr0b2 is proved to regulate the transcriptional activity of Runx2 and promote osteoblast differentiation and bone formation (Jeong et al., 2010). MiR-23a cluster mediated osteogenic differentiation regulation by modulating the transforming growth factor beta (TGF-β) signaling pathway after targeting Prdm16 (Zeng et al., 2017). Msx1 is reported to enhance the osteogenic differentiation of multipotent muscle satellite cells and human dental pulp stem cells (Goto et al., 2016; Ding et al., 2017). Pitx1, which inhibits Wnt pathway and the self-renewal of mesenchymal stem cells, induced senile osteoporosis in mice (Karam et al., 2019). The GO terms of this network, including calcium ion binding, hormone activity, and extracellular space, regulated the pathological process of OP. KEGG pathway analyses show that this network is involved in PPAR signaling pathway, AMPK signaling pathway, and lipolysis regulation in adipocyte and adipocytokine signaling pathway. These results indicate that the lncRNA NONMMUT096150.1 might directly or indirectly regulate the adipogenesis of BMSCs. Further work needs to be carried out to explore the lncRNA’s biological functions.

This study has some limitations. First, the expression level of key DEmRNAs needs to be verified by qRT-PCR. Second, the prognostic value of key lncRNAs and genes and their role in the development of PMOP need to be further explored through experiments. In future research, we will further explore the relationship between the expression levels of key lncRNAs and genes and the prognosis of OP patients, and the specific regulatory mechanisms of key lncRNAs and genes.

In this study, we first systematically identified OP-associated lncRNAs in OVX mice and constructed lncRNAs/mRNAs coexpression network. The lncRNAs played crucial roles in the differentiation of BMSCs during OP. The NONMMUT096150.1 was verified by network and bioinformatics to be a key lncRNA in OP, regulating the adipogenesis of BMSCs. The data further deepened our understanding of lncRNAs along with their functions in the pathogenesis of OP. Nevertheless, more and more verification is needed to explore the detailed mechanism of lncRNAs under OP.

## Data Availability Statement

The datasets presented in this study can be found in online repositories. The names of the repository/repositories and accession number(s) can be found below: https://www.ncbi.nlm.nih.gov/sra/PRJNA737522, BioProject accession: PRJNA737522.

## Ethics Statement

The animal study was reviewed and approved by the animal experimental protocols got the approval of the Animal Care and Experiment Committee of Fudan University.

## Author Contributions

HG, KZ, and XY: conception and design. HG, ZH, and JX: development of methodology. HG and GC: sample collection. ZH and CB: analysis and interpretation of data. HG, KZ, and JX: writing, review, and revision of the manuscript. All authors contributed to the article and approved the submitted version.

## Conflict of Interest

The authors declare that the research was conducted in the absence of any commercial or financial relationships that could be construed as a potential conflict of interest.

## Publisher’s Note

All claims expressed in this article are solely those of the authors and do not necessarily represent those of their affiliated organizations, or those of the publisher, the editors and the reviewers. Any product that may be evaluated in this article, or claim that may be made by its manufacturer, is not guaranteed or endorsed by the publisher.

## Footnotes

1
http://bioconductor.org/packages/release/bioc/html/DESeq.html
